# Direct Aspiration Thrombectomy in the Management of Procedural Thromboembolic Complications Related to Endovascular Brain Aneurysm Treatment

**DOI:** 10.3390/medicina60071034

**Published:** 2024-06-24

**Authors:** Damljan Bogicevic, Filip Vitosevic, Svetlana Milosevic Medenica, Vladimir Kalousek, Marjana Vukicevic, Lukas Rasulic

**Affiliations:** 1 Special Hospital for Cerebrovascular Diseases “Sveti Sava”, 11000 Belgrade, Serbia; 2 Faculty of Medical Sciences, University of Kragujevac, 34000 Kragujevac, Serbia; 3 Faculty of Medicine, University of Belgrade, 11000 Belgrade, Serbia; 4 University Clinical Hospital Center ‘Sisters of Mercy’, 10000 Zagreb, Croatia; 5 Clinic for Neurosurgery, University Clinical Center of Serbia, 11000 Belgrade, Serbia

**Keywords:** endovascular treatment, brain aneurysm, neuroradiology, mechanical thrombectomy, stroke management, managing complications

## Abstract

Despite growing evidence over the last few years of the efficacy and safety of direct thrombus aspiration using a large bore distal access catheter as a type of mechanical thrombectomy procedure in acute stroke large-vessel occlusion patients, the experience and evidence of this technique for managing thromboembolic complications in endovascular aneurysm treatment is still limited and little research is available regarding this topic. We present a case of a thromboembolic occlusion of the left middle cerebral artery during the preprocedural angiograms of a large and fusiform left internal carotid artery aneurysm. This complication was successfully managed by navigating an already-placed distal access catheter intended for support during the opening of the flow-diverting stent; therefore, the thrombus was manually aspirated for two minutes, and Thrombolysis in Cerebral Infarction (TICI) scale 3 flow was restored. This case should encourage the use of a distal access catheter, already placed for aneurysm treatment, to perform zero-delay direct thrombus aspiration as a rescue approach for thromboembolic complications during endovascular treatments.

## 1. Introduction

Although numerous efforts have been invested in reducing the incidence of periprocedural complications related to endovascular treatment of intracranial aneurysms, thromboembolism is still a major complication alongside intraprocedural aneurysmal perforation. It can cause partial or total occlusion of arterial branches and may result in transient or permanent neurologic deficit. The main origins of thrombus are reported to be clot formation in the guiding catheter, the clot formation in the parent blood vessel due to the induced vasospasm, the presence of intravascular foreign material itself, the electric current used for the detachment of the material, as well as clots from large (sometimes partially thrombosed) slow contrast filling aneurysms [1,2,3]. Protective protocols, such as preoperative dual antiplatelet therapy and systemic intraprocedural heparinization, are utilized to prevent this threatening event.

Many studies have suggested the use of glycoprotein (GP) IIb/IIIa inhibitors or fibrinolytic agents as a rescue therapy for intraprocedural thrombus formation during endovascular aneurysm treatments [3,4,5,6]. While the intra-arterial application of these agents is considered to be a treatment of choice for an acute intraprocedural thromboembolism, the efficacy and safety of a direct aspiration mechanical thrombectomy (MT) as a rescue method for thromboembolic occlusions has rarely been used and reported in the literature. In recent studies, a direct aspiration MT technique was shown to be effective, safe, comparable in clinical efficacy compared to thrombus removal via the stent retriever, and even had a significantly shorter procedure time [7,8,9].

We present a case of a thromboembolic occlusion of the left middle cerebral artery (MCA) during the preprocedural angiograms of a large and fusiform left internal carotid artery (ICA) aneurysm, successfully managed by direct thrombus aspiration using a distal access catheter (DAC) and followed by the opening of the flow-diverting stent.

## 2. Case Report

**Figure 1:**
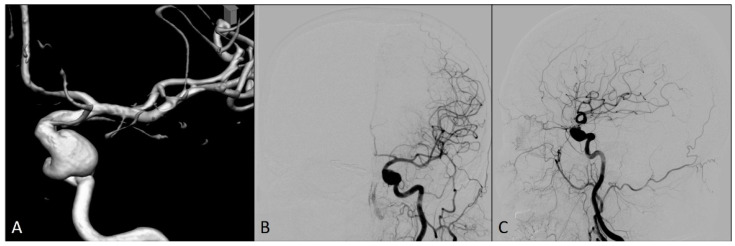
Preprocedural diagnostic digital subtraction angiography imaging of the left internal carotid artery: (**A**) 3D view; (**B**) antero-posterior view; and (**C**) lateral view.

A 54-year-old woman was admitted to our hospital for an elective endovascular treatment of two intracranial left ICA aneurysms (Figure 1). She was previously operated on due to the ruptured right ICA aneurysm 10 years ago, when a neurosurgical clip was placed over the neck of this aneurysm. In regular computed tomography angiography (CTA) controls, an additional two left ICA aneurysms were identified, and the patient was then followed up until it was decided to treat these aneurysms.

**Figure 2:**
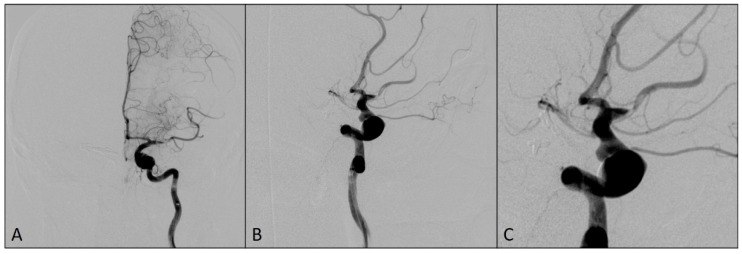
Angiograms showing a thromboembolic occlusion of the M1 and M2 segments of the left middle cerebral artery after the placement of DAC: (**A**) antero-posterior view; (**B**) oblique view; and (**C**) magnified oblique view.

The patient has a history of hypertension. Modified Rankin Scale (mRS) at hospital admission was 0. The patient was prepared for the intervention with the dual antiplatelet therapy since the placement of flow-diverting stent to cover the origins of these left ICA aneurysms was intended. Daily doses of 150 mg of clopidogrel and 100 mg of aspirin were orally administered for four days before the procedure and the platelet function was tested using VerifyNow P2Y12 assay (Accumetrics, San Diego, CA, USA) on the day before the procedure. The test showed a good response to P2Y12 inhibitors as well as sensitivity to aspirin. The procedure was performed with the patient under general anesthesia. The administration of an intravenous bolus dose of 5000 IU of heparin, as systemic anticoagulation, was started after the placement of a long sheet 6F 088 Neuron Max (Penumbra Inc, Alameda, CA, USA) into the left ICA and was followed by a bolus dose of 1000 IU of heparin each hour. On the final preprocedural angiograms, after the placement of DAC Sofia5F (MicroVentionEurope, Saint-Germain-en-Laye, France), the thromboembolic occlusion of the M1 and M2 segments of the left MCA was observed (Figure 2).

**Figure 3:**
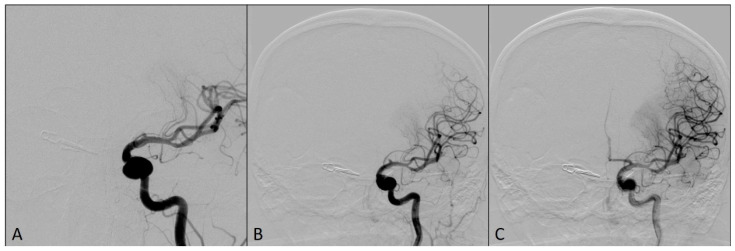
Angiograms after the flow was restored in left middle cerebral artery territory: (**A**) Magnified antero-posterior view; (**B**) arterial phase, antero-posterior view; (**C**) capillary phase, antero-posterior view.

This complication was managed using an already-placed DAC through a long sheath to avoid having a time delay in the placement of the larger bore DAC. The DAC Sofia5F was placed in the M1 segment of the left MCA with the support of the Rebar18 microcatheter (ev3, Irvine, CA, USA) and the 0.014 Avigo guide wire (ev3, Irvine, CA, USA), so that the tip of the catheter was in contact with the clot. The thrombus was manually aspirated for two minutes, and Thrombolysis in Cerebral Infarction scale 3 flow was restored in the left MCA territory (Figure 3).

**Figure 4:**
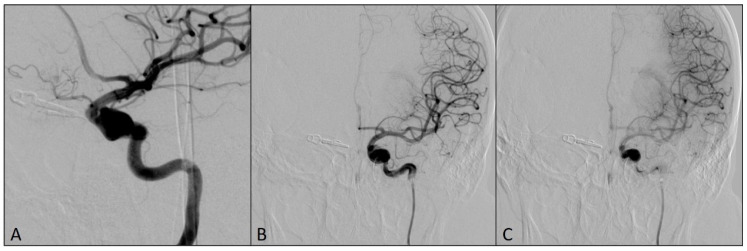
Final postprocedural angiograms after the placement of the flow-diverting stent over the origins of left ICA aneurysms: (**A**) oblique view; (**B**) arterial phase, antero-posterior view; (**C**) capillary phase, antero-posterior view (notice the stasis of the contrast medium in the aneurysm sacs—flow-diverting effect).

Then, DAC was pulled back into the left ICA, with the tip located proximally to the fusiform aneurysm. The microcatheter Phenom 0.027 (Medtronic, Irvine, CA, USA), intended for the delivery of the flow-diverting stent, was placed distally from the origin of both ICA aneurysms and the flow-diverting stent Pipeline (Medtronic, Irvine, CA, USA) was placed to cover the origins of both aneurysms (Figure 4).

A follow-up CT scan on the day after the procedure showed no pathologic density changes. The patient was discharged from the hospital three days after the procedure, without any neurological symptoms and an mRS score of 0. According to the guidelines, conventional secondary prevention therapy of stent thrombosis was added to the patient’s standard therapy.

**Figure 5:**
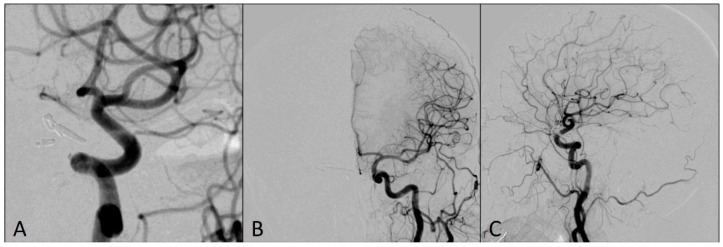
Left ICA DSA angiograms at six-month follow-up showing completely occluded aneurysms: (**A**) magnified oblique view; (**B**) antero-posterior view; (**C**) lateral view.

Follow-up angiograms six months after the procedure showed that both aneurysms were completely occluded, without any residual sac filling, while MCA territory was completely patent (Figure 5). The patient’s mRS score at six-month follow-up was 0.

## 3. Discussion

Thromboembolic complications during the endovascular treatment of intracranial aneurysms are still a major burden, occurring in about 2.5–28% of patients [1,6,10,11]. In the study by van Rooij WJ et al. about complications during the endovascular treatment of ruptured intracranial aneurysms, thromboembolic complications occurred in 4.7%, accounting for 1.9% mortality and 2.8% morbidity [12,13]. A relatively high incidence of thromboembolism during endovascular treatment of ruptured intracranial aneurysms compared to unruptured ones can be associated with the limited use of preoperative antiplatelet therapy and the hypercoagulable status in patients with ruptured aneurysms [11]. Despite heparinization, both systemic and through the guiding catheter and the microcatheter, embolic events can occur during the treatment from several sources; they include damage of fragile plaques, iatrogenic dissection, air bubbles, and thrombus or fresh clots formation within the aneurysm and catheters [13,14]. In our case, we noticed slow contrast filling of the aneurysm fundus bottom, with the stagnation of the contrast medium throughout the venous phase. This part of the fundus could be the origin of our clot, which eventually migrated distally during the injection of contrast medium. Clot formation inside an aneurysm sac can occur as a natural response to repair the weakened artery wall. When an aneurysm forms, the artery wall becomes thin and bulges out, creating a potential site for rupture. The defense mechanism is to form a clot at the site of injury to prevent potential bleeding. The clot may partially or completely fill the sac, reducing the risk of rupture by providing structural support to the weakened arterial wall [15]. We should also reiterate that our patient received dual antiplatelet therapy since the placement of flow-diverting stent was planned. This could advocate for abandoning the theory of clot formation inside the catheters and favor the theory of clot migration from the bottom of the fundus of the large and fusiform aneurysm.

The usual treatment for an acute thromboembolism during the endovascular treatment of intracranial aneurysms includes the use of intra-arterial (IA) fibrinolytic agents or GP IIb/IIIa inhibitors [1,2,6,11]. According to recent reports, and in our experience, these inhibitors have been used more often and have provided better outcomes [6,11,16]. Although it has been demonstrated that the rescue therapy with GP IIb/IIIa inhibitors is associated with better outcomes in both ruptured and unruptured aneurysms, they are not easily reversed, resulting in increased hemorrhagic risk, especially in our case, where the patient was already prepared with the dual antiplatelet therapy for the procedure [6]. A study by Cho YD et al. found that the 10.5% of their patients received an IA GP IIb/IIIa inhibitor during the treatment of ruptured intracranial aneurysms, and although a high rate of recanalization (87.2%) was obtained in these patients, intracerebral hematoma occurred in 5.1% and cerebral infarction developed in 20.5% of these patients [4]. Ahn et al. observed hemorrhagic complications in two of five patients who had received an IA GP IIb/IIIa inhibitor before MT, whereas none were found in patients treated by MT only [11].

One of our major concerns regarding possible MT with a retrievable stent in our case was the injury of the fusiform aneurysm by stent struts during the retrieval procedure and potential distal thromboembolism. Detachment of the retrievable device has also been proposed, but it is associated with higher rates of symptomatic intracranial hemorrhage, poorer outcome, and higher mortality rate [13,17].

Despite the growing evidence over the last two years for the efficacy and safety of direct thrombus aspiration with a large bore distal access catheter as a type of MT procedure in acute stroke large-vessel occlusion patients, the experience and evidence of this technique as a solution for managing thromboembolic complications during endovascular aneurysm treatment is still limited and little research is available regarding this topic.

In a recent multicenter study, the aspiration first pass technique (ADAPT) achieved a high recanalization rate (78%) in acute ischemic stroke patients, similar to the stent retrievers [13,18]. A current report indicates that ADAPT and the stent retriever technique are comparable in clinical outcomes [7,9]. Shallwani et al. showed that DAC used for ADAPT was successfully positioned near the occlusion site in 92.7% of cases, and good reperfusion results were achieved in 73.3% of cases [9,19]. Zhang et al. reported that patients treated with ADAPT had higher rates of receiving additional therapy such as stent-assisted MT if ADAPT first pass failed. However, in the future, ADAPT may become more widespread in clinical use, due to its usability, less harm to vessel walls, and lower costs [20]. A direct thrombus aspiration without the necessity to pass through the occlusion and the possibility of not using adjunctive devices such as stent-retrievers are the main advantages of this technique.

Our patient had a thromboembolic occlusion of the left MCA, in the same territory and distally to the large and fusiform aneurysm intended for treatment. This prompted us to contemplate the thrombus aspiration technique using an already-placed distal access catheter for zero-delay aspiration. Advancing DAC through the fusiform aneurysm can be considered a high risk for procedure-related rupture of the aneurysm. Patients with saccular aneurysms and small neck diameter should represent more convenient and safer cases for DAC navigation. We used a microcatheter and a guidewire as a support to advance the DAC proximal to the occlusion site in the left MCA. The DAC was advanced without any complications, and the thrombus was successfully manually aspirated. Therefore, we continued the initial procedure and carried out the placement of the flow-diverting stent to cover the origins of one large fusiform and one medium-sized aneurysm of the left ICA. On the angiograms after the deployment of flow-diverting stent, the stasis of the contrast medium (flow-diverting effects) was observed in the aneurysm sacs, while all vessels and vascular branches remained patent.

## 4. Conclusions

In our case, we achieved complete recanalization of procedural thromboembolic occlusion of the left MCA and its branches, without any ischemic or hemorrhagic events, followed by good clinical and neurological outcomes. Managing thromboembolic complications during endovascular treatment of intracranial aneurysms could increase the risk of aneurysmal rupture and should be performed very carefully. This case should encourage the use of DAC that is already placed for support of an aneurysm treatment to perform zero-delay direct thrombus aspiration as a rescue treatment in thromboembolic complications.

## Data Availability

No new data were created or analyzed in this study. Data sharing is not applicable to this article.
